# Supplementary material for: Current Challenges of Methylation-Based Liquid Biopsies in Cancer Diagnostics
Source: Cancers (Basel). 2024 May 24;16(11):2001. doi: 10.3390/cancers16112001 (PMC11171112; doi:10.3390/cancers16112001)
Supplement: Supplementary file 1 [file cancers-16-02001-s001.zip › cancers-3001558-supplementary.pdf]

Supplementary material:

Table S1. Overview of methylation detection methods in cfDNA listed with their characteristic, major advantages and disadvantages in practical use. .

Method	Characteristic	Major advantage	Major disadvantage
Bisulfite Conversion followed by PCR or Sequencing	Converts unmethylated cytosine residues to uracil, leaving methylated cytosines unchanged. Can be combined with various downstream methods.	Can detect methylation patterns accurately.	Requires careful optimization to minimize DNA degradation and bias introduced during bisulfite treatment.
Methylation-Specific PCR (MSP)	Selective amplification of methylated or unmethylated DNA sequences using specific primers.	Relatively simple and cost-effective method.	Requires prior knowledge of methylation status to design specific primers. Can be prone to PCR bias and optimization challenges.
Quantitative Methylation-Specific PCR (qMSP)	Incorporates real-time quantitative PCR to measure methylation levels.	Provides quantitative data on methylation levels.	Similar limitations as MSP, including primer design challenges and potential for PCR bias.
Pyrosequencing	Allows quantitative analysis of DNA methylation at specific CpG sites.	Provides quantitative, single-nucleotide resolution data.	Costly instrumentation and reagents. Sequence context can affect assay accuracy.
Methylated DNA Immunoprecipitation (MeDIP)	Immunoprecipitates methylated DNA fragments for downstream analysis.	Can analyze large genomic regions for methylation patterns.	May suffer from antibody specificity issues. Requires additional steps for downstream analysis.
Methylation-Sensitive Restriction Enzymes (MSRE) Digestion	Digests methylated DNA at specific recognition sites.	Simple and cost-effective method.	Limited by recognition sites of available enzymes. Cannot provide single-nucleotide resolution data.
Mass Spectrometry-Based Methods	Quantitatively analyzes DNA methylation at specific CpG sites.	High throughput and quantitative measurement of methylation levels.	Expensive instrumentation and specialized expertise required. Limited by the number of CpG sites that can be analyzed simultaneously.
Whole-Genome Bisulfite Sequencing (WGBS)	Provides comprehensive, genome-wide DNA methylation profiling at single-nucleotide resolution.	Offers comprehensive coverage of methylation patterns.	High sequencing and computational costs. Requires high-quality DNA and extensive data analysis.
Reduced Representation Bisulfite Sequencing (RRBS)	Targets CpG-rich regions for reduced sequencing costs while maintaining genome-wide coverage.	Reduces sequencing costs compared to WGBS.	Limited coverage of non-CpG methylation.
Nanopore Sequencing	Directly detects DNA methylation by measuring changes in electrical currents as DNA passes through nanopores.	Long reads and real-time data analysis.	Higher error rates compared to other sequencing technologies. Costly instrumentation and reagents.
Single-Molecule Real-Time Sequencing (SMRT Sequencing)	Monitors kinetic signatures of DNA base modifications for high-resolution methylation data.	Provides long-read, high-resolution methylation data.	High error rate and cost. Requires high DNA input and specialized expertise for data analysis.
Infinium Methylation Arrays	Enables high-throughput, genome-wide methylation profiling via hybridization-based methods.	High throughput and coverage of CpG sites across the genome.	Limited resolution for specific CpG sites. Data interpretation may be complex due to probe design and background noise.
